# Antimicrobial Activity of Essential Oil of *Baccharis dracunculifolia* DC (Asteraceae) Aerial Parts at Flowering Period

**DOI:** 10.3389/fpls.2019.00027

**Published:** 2019-01-29

**Authors:** Luciane Neris Cazella, Jasmina Glamoclija, Marina Soković, José Eduardo Gonçalves, Giani Andrea Linde, Nelson Barros Colauto, Zilda Cristiani Gazim

**Affiliations:** ^1^Graduate Program in Biotechnology Applied to Agriculture, Chemistry Laboratory of Natural Products, Paranaense University, Umuarama, Brazil; ^2^Institute for Biological Research “Siniša Stanković”, University of Belgrade, Belgrade, Serbia; ^3^Graduate Program in Clean Technologies and Institute of Science, Technology and Innovation of Cesumar University Center, Maringá, Brazil

**Keywords:** alecrim-do-campo, vassourinha, GC–MS, inhibiting activity, *Staphylococcus aureus*, *Bacillus cereus*, *Pseudomonas aeruginosa*

## Abstract

*Baccharis dracunculifolia* DC (Asteraceae) is a Brazilian native bush tree, and its leaf essential oil has been reported to possess some biological activities, but the antimicrobial activity of its aerial part essential oil at the flowering period is unknown or little studied, mainly against agents that cause foodborne diseases. Thus, this study aimed to determine the chemical composition and evaluate the antimicrobial activity of the essential oil of *B. dracunculifolia* aerial part at flowering period. This essential oil was obtained by hydro distillation and its chemical composition was determined by gas chromatography coupled with mass spectrometry (GC–MS). The minimum inhibitory concentration, minimum bactericidal concentration, and minimum fungicidal concentration of the essential oil were evaluated against eight bacteria and eight fungi using 96-well microtiter plates. The essential oil yield was 1.8 ± 0.07%, and spathulenol (27%) and *trans*-nerolidol (23%), both oxygenated sesquiterpenes, were the major compounds found among 30 chemical constituents identified. The essential oil presented bacteriostatic and bactericidal activities, mainly against *Staphylococcus aureus*, *Bacillus cereus* and *Pseudomonas aeruginosa*, and also fungistatic and fungicidal activities. However, its antibacterial activity was more effective than the antifungal one by using the essential oil at lower concentrations. Essential oil of *B. dracunculifolia* may be a potential alternative for food applications in order to reduce synthetic chemicals in a more sustainable food industry.

## Introduction

*Baccharis dracunculifolia* DC (Asteraceae), known as “alecrim-do-campo” (rosemary-of-the-field) and “vassourinha” (small broom) in Brazil (Barroso, 1976), a perennial woody bush tree that can reach up to around 2 m of height with alternate spike lanceolate leaves (Borges and Forzza, 2008), 1 to 1.5-mm long achene (Barroso, 1976), dioicous with male and female inflorescences in separate plants (Park et al., 2004), is native to biomes of Brazilian Cerrado, Atlantic Rainforest and Pampas. It is reported as the main source of propolis and bee honey production, followed by *Araucaria angustifolia* and *Eucalyptus citriodora* in Brazil (Sforcin et al., 2012).

Hydroalcoholic and methanolic extracts of *B. dracunculifolia* presented antiparasitic, immunomodulatory, anti-inflammatory, antitumor, and antiulcer activities (Sforcin et al., 2012). Methyl linoleate, caryophillene, and *trans*-nerolidol were isolated from the hexanic fraction from the hydroalcoholic extract of aerial parts of *B. dracunculifolia* and reported to have action against *Paracoccidioides brasiliensis* (clinical isolates) (Johann et al., 2012). Also, *B. dracunculifolia* leaf essential oil was reported to have antifungal action against *Candida albicans* (Pereira et al., 2011), fungal phytopathogens such as *Rhizoctonia solani*, *Sclerotium rolfsii*, and *Sclerotinia minor* (Fonseca et al., 2015), and antibacterial action against *Mycobacterium* sp. (Machado et al., 2015) and *Streptococcus mutans* (Pereira et al., 2016).

Despite of some reports on the antimicrobial activity of *B. dracunculifolia*, mainly related to its antifungal activity, the antimicrobial activity spectrum of *B. dracunculifolia* essential oil in the flowering phenophase and against agents causing foodborne diseases is unknown or little studied. Food spoilage is a serious widely neglected problem, mainly by mycotoxins in grain storage due to poor harvesting practices, inappropriate drying, handling, packaging, storage, and transport conditions (Bhat et al., 2010). The utilization of synthetic chemicals has increased microorganism resistance, mostly against antibiotics, and the search for alternative agents to control microorganisms is therefore necessary (Rossolini et al., 2014; Ventola, 2015). An alternative to reduce synthetic chemicals is the search for antimicrobials from medicinal plants (Anyanwu and Okoye, 2017) to extend shelf life and combat foodborne pathogens. Thus, the aim of this study was to determine the chemical composition and evaluate the antimicrobial activity of *B. dracunculifolia* aerial part essential oil for potential applications to preserve food by non-synthetic compounds.

## Materials and Methods

### Plant Materials

*Baccharis dracunculifolia* aerial parts (leaves and flowers) were harvested in Guaraniaçu, Brazil, at the coordinates 25°08′05, 47″ S and 52°53′49, 23″W, 800 m of altitude, between 7 and 8 O’clock in the morning, in April, 2016, during the flowering phenophase at the ratio of 8:1 (bud flower:open flower) in the inflorescence. The sample was identified by Dr. Gustavo Heiden and an exsiccate was deposited in the collection of the Herbarium of the State University of West Paraná, campus of Cascavel, PR, Brazil, under the registration number UNOP-8655.

### Essential Oil Extraction

*Baccharis dracunculifolia* aerial parts were air dried under the shaded for 1 week. The essential oil was extracted from 200 g of dried aerial parts in 2 L of distilled water by hydrodistillation in a modified Clevenger apparatus for 2 h (Miranda et al., 2016) and stored at 4°C (Pereira et al., 2016).

The essential oil yield (%) was calculated by mass (g) of essential oil per mass (g) of the dried aerial parts of the plant. The essential oil absolute density was determined in graduated capillaries (5.0 μL) and calculated by mass (g) per volume (mL) at 20°C. The refraction index was determined using an Abbe refractometer (RL3 model) which was calibrated with distilled water (refraction index of 1.3330) at 20°C (Brasil, 1988).

### Chemical Characterization

The essential oil chemical identification was carried out by GC–MS (Agilent 19091J-433) equipped with an HP-5MS capillary column (30 m × 0.25 mm × 0.25 μm), with initial temperature from 40°C (2 min) to 230°C (3°C/min), and kept at this temperature for 20 min. Helium was utilized as the carrier gas at the linear speed of 1 mL/min up to 300°C, and pressure release of 56 kPa. The injector temperature was 250°C; the injection volume was 1 μL; the injection occurred in split mode (20:1). The temperatures of the transfer line, ion source, and quadrupole were 285, 230, and 150°C, respectively. Mass spectrometry was obtained with a scan range of 40 to 550 *m*/*z* with solvent delay of 3 min, compounds were identified based on comparison of their retention indices obtained by a homologous series of *n*-alkane standard (C7–C28), and electron ionization mass spectra were compared with the Wiley 275 library spectra (Adams, 2012).

### Antibacterial Activity

The antibacterial activity of *B. dracunculifolia* essential oil was tested against eight bacterium species: Gram-positive *Bacillus cereus* Frankland and Frankland (clinical isolate), *Listeria monocytogenes* (Murray et al.) Pirie (NCTC 7973), *Micrococcus flavus* (ATCC 10240), and *Staphylococcus aureus* subsp. *aureus* Rosenbach (ATCC 6538) bacteria, and Gram-negative *Enterobacter cloacae* (Jordan) Hormaeche and Edwards (clinical isolate), *Escherichia coli* (Migula) Castellani and Chalmers (ATCC 35218), *Pseudomonas aeruginosa* (Schroeter) Migula (ATCC 27853), and *Salmonella enterica* subsp. *enterica* (ex Kauffmann and Edwards) Le Minor and Popoff serovar Typhimurium (ATCC 13311). The microorganisms were from the Mycology Laboratory of the Institute for Biological Research “Siniša Stanković”, University of Belgrade, Serbia.

The antibacterial assay was done by microdilution method (Clinical and Laboratory Standards Institute [CLSI], 2012; Tsukatani et al., 2012) utilizing 96-well microtiter plates to determine the minimum inhibitory concentration (MIC) and minimum bactericidal concentration (MBC). The bacterial suspensions were adjusted with sterile saline solution until the concentration of 1.0 × 10^5^ CFU/mL. The inoculum was prepared daily and stored at 4°C until its utilization. The inoculum was cultivated in solid medium to verify the absence of contaminations, and for validation. *B. dracunculifolia* essential oil was dissolved in a 5% dimethyl sulfoxide solution (Merck KGaA, Germany) containing 0.1% of polysorbate-80 (1 mg/mL) and added to Luria-Bertani (100 μL) medium with bacterial inoculum (1.0 × 10^4^ CFU/well) to reach the desired concentrations. The microplates were incubated in a rotary agitator (160 rpm), for 24 h, at 37°C. The lowest concentration without visible of the microbial biomass growth under the optical microscope were defined as the concentrations that completely inhibited bacterial growth.

Minimum bactericidal concentration was determined by 2 μL serial sub cultivation in microtiter plates containing 100 μL of broth per well and incubation during 24 h. The lowest concentration without visible microbial biomass growth under optical microscope was defined as MBC, indicating the death of 99.5% of the original inoculum. The optical density for each well was measured in a 655 nm wavelength with a Microplate Manager 4.0 (Bio-Rad Laboratories) and compared to a blank one (broth medium with diluted essential oil) and positive control. Streptomycin (Sigma P7794) and ampicillin (Panfarma, Belgrade, Serbia) were utilized as positive controls (1 mg/mL in sterile saline solution). A solution of 5% dimethyl sulfoxide was utilized as negative control.

### Antifungal Activity

The antifungal activity of *B. dracunculifolia* essential oil was tested against eight fungi: *Aspergillus fumigatus* Fresenius (ATCC 1022), *A. niger* van Tieghem (ATCC 6275), *Aspergillus versicolor* (Vuillemin) Tiraboschi (ATCC 11730), *A. ochraceus* Wilhelm (ATCC 12066), *Penicillium funiculosum* Thom (ATCC 8725), *Penicillium ochrochloron* Biourge (ATCC 9112), *Penicillium verrucosum* var. *cyclopium* (Westling) Samson, Stolk & Hadlok (food isolate), and *Trichoderma viride* Pers. (IAM 5061). The microorganisms were from the Mycology Laboratory of the Institute for Biological Research “Siniša Stanković”, University of Belgrade, Serbia.

The fungi were kept in malt extract agar, and the cultures were stored at 4°C and subcultivated once a month (Booth, 1971). A modified microdilution technique was utilized to investigate the antifungal activity (Hänel and Raether, 1988; Espinel-Ingroff, 2001). The fungal spores were washed with sterile saline solution at 0.85% containing polysorbate-80 (0.1%). The spore suspension was adjusted with sterile saline solution to a concentration of 1.0 × 10^5^ in a final volume of 100 μL per well. The inoculums were stored at 4°C for posterior utilization. The inoculum dilutions were cultivated in malt extract agar to verify the absence of contamination and validate each inoculum.

Minimum inhibitory concentration was determined by serial dilution technique using 96-well microtiter plates. The essential oil was dissolved in 5% dimethyl sulfoxide solution (Merck KGaA, Germany), containing 0.1% of polysorbate-80 (1 mg/mL), and added to a malt extract cultivation medium with inoculum. The microplates were incubated in a rotary agitator (160 rpm) for 72 h at 28°C. The lowest concentrations without visible microbial biomass growth under optical microscope were defined as the concentrations that completely inhibited fungal growth.

The minimum fungicidal concentration (MFC) was determined by a 2 μL serial sub cultivation of the tested compound dissolved in a cultivation medium, and inoculated during 72 h in microtiter plates containing 100 μL of broth per well and with incubation for 72 h at 28°C. The lowest concentration without visible biomass concentration was defined as MFC indicating the death of 99.5% of the original. The commercial fungicides bifonazole (Srbolek, Belgrade, Serbia) and ketoconazole (Zorkapharma, Šabac, Serbia) were used as positive controls (1–3500 μg/mL).

### Statistical Analysis

The antimicrobial tests were carried out in duplicate and replicated three times. The results were expressed in values of arithmetical average ± standard deviation and analyzed by analysis of unidirectional variance (ANOVA), followed by Tukey’s HSD (honestly significant difference) test with α = 0.05 to determine statistical differences. The analysis was done by Statistical Package for the Social Sciences, v. 22.0.

## Results

*Baccharis dracunculifolia* essential oil was colorless with a characteristic honey odor. The essential oil yield was 1.8 ± 0.07%, density was 1.01 ± 0.001 g/mL, and the refraction index 1.4970. Thirty constituents were identified in the essential oil and their main classes were oxygenated sesquiterpenes (60.8%), hydrocarbon sesquiterpenes (22.9%), and hydrocarbon monoterpenes (9.6%) (Table 1). The major compounds were spathulenol (27.4%) and *trans*-nerolidol (23.1%), followed by heptacosane (6.0%), β-pinene (5.6%), bicyclogermacrene (4.7%), *trans*-caryophyllene (4.6%), germacrene D (4.5%) and α-muurolol (4.1%), representing 80% of the compounds (Table 1).

**Table 1:** Chemical composition of *Baccharis dracunculifolia* aerial part essential oil during flowering period.

Peak	Compounds^a^	Area (%)	RI^b^	Identification method
	**Hydrocarbon monoterpenes**	**(9.58)**		
1	α-Pinene	1.30	932	a, b, c
2	β-Pinene	5.41	974	a, b, c
3	Limonene	2.87	1024	a, b, c
	**Oxygenated monoterpenes**	**(0.31)**		
4	α-Terpineol	0.16	1186	a, b, c
5	Myrtenol	0.15	1194	a, b, c
	**Hydrocarbon sesquiterpenes**	**(22.87)**		
6	δ-Elemene	0.33	1335	a, b, c
7	α-Copaene	0.29	1374	a, b, c
8	β-Bourbonene	0.07	1387	a, b, c
9	β-Elemene	0.57	1389	a, b, c
10	α-Gurjunene	0.15	1409	a, b, c
11	*Trans*-Caryophyllene	4.60	1417	a, b, c
12	β-Ylangene	0.42	1419	a, b, c
13	β-Copaene	0.34	1430	a, b, c
14	α-Humulene	0.84	1452	a, b, c
15	*allo*-Aromadendrene	1.28	1458	a, b, c
16	γ-Muurolene	0.31	1478	a, b, c
17	Germacrene D	4.49	1484	a, b, c
18	Bicyclogermacrene	4.70	1500	a, b, c
19	α-Muurolene	0.68	1500	a, b, c
20	γ-Cadinene	0.49	1513	a, b, c
21	δ-Cadinene	3.31	1522	a, b, c
	**Oxygenated sesquiterpenes**	**(60.80)**		
22	*Trans*-Nerolidol	23.06	1564	a, b, c
23	Spathulenol	27.43	1577	a, b, c
24	Viridiflorol	2.01	1592	a. b. c
25	*Epi*-α-Cadinol	1.46	1638	a, b, c
26	α-Muurolol	4.08	1644	a, b, c
27	Cedren-13-ol, 8-	0.83	1688	a, b, c
28	Murolan-3,9(11)-diene-10-peroxy	1.93	1729	a, b, c
	**Oxygenated diterpenes**	**(0.11)**		
29	Phytol	0.11	1942	a, b, c
	**Other compounds**	**(6.02)**		
30	Heptacosane	6.02	2700	a, b, c
	**Total identified**	**(99.69)**		

*^a^Compounds listed according to elution order from HP-5MS; ^b^calculated retention index (RI) utilizing n-alkanes c_7_ to c_26_ in capillary column (HP-5MS); ^c^identification based on the comparison of mass spectra from Wiley 275 libraries.*

**FIGURE 1:**
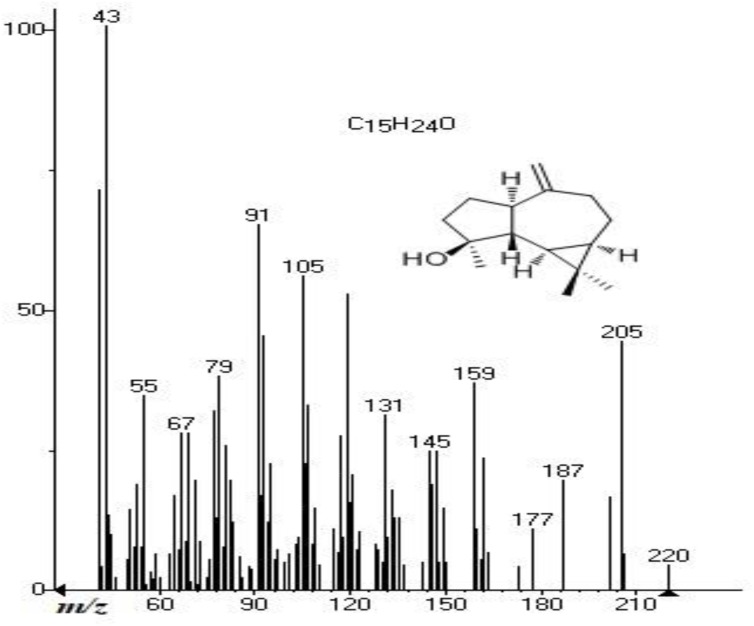
Spathulenol electron ionization mass spectrum (*m/z* = 220.18) obtained by gas chromatography-mass spectrometry of *Baccharis dracunculifolia* aerial part essential oil.

Spathulenol is the major compound found in our study and the mass spectrum was obtained by GC–MS (Figure 1). This compound is produced by oxidative cyclization of bicyclogermacrene (Tran and Cramer, 2014). Spathulenol is a sesquiterpene component of essential oils in several aromatic species (Nascimento et al., 2018) and reported with antimicrobial (Tan et al., 2016), antiproliferative, anti-inflammatory, and immunomodulatory (Ziaei et al., 2011) activities.

**FIGURE 2:**
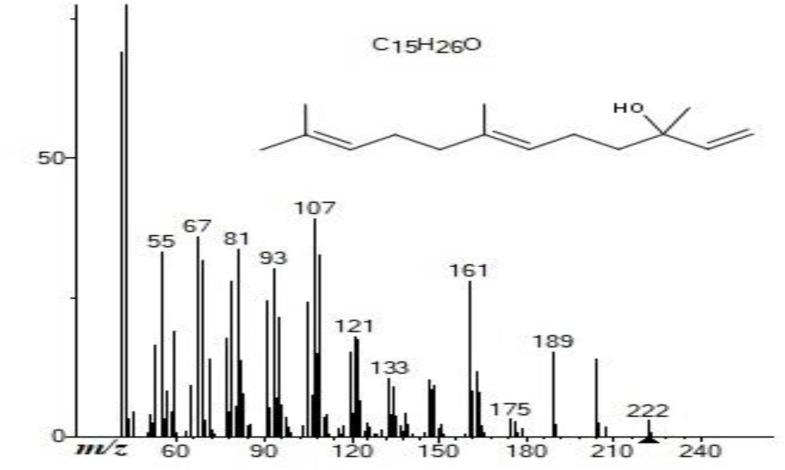
*Trans*-Nerolidol electron ionization mass spectrum (*m/z* = 222.19) obtained by gas chromatography-mass spectrometry of *B. dracunculifolia* aerial part essential oil.

*Trans*-nerolidol (3,7,11-trimethyl-1,6,10-dodecatrien-3-ol) is the second major compound of our study, and the mass spectrum was obtained by GC–MS (Figure 2). It is also known as peruviol, and is a naturally occurring sesquiterpene alcohol found in essential oil of several plants with floral odor. This compound has four different isomeric forms, which consist of two enantiomers and two geometric isomers: *cis*- and *trans*-nerolidol (Chan et al., 2016).

MIC values of the essential oil for bacteria varied from 0.50 to 12.65 mg/mL and the controls streptomycin and ampicillin ranged from 0.04 to 0.75 mg/mL (Table 2). MBC values of the essential oil varied from 1.50 to 16.87 mg/mL and for the controls streptomycin and ampicillin from 0.10 to 1.20 mg/mL (Table 2). *S. aureus*, *B. cereus* and *P. aeruginosa* were the most susceptible species to essential oil with MIC of 0.5, 1.1, and 1.05 mg/mL, respectively, and MBC of 2.1, 1.5, and 2.1 mg/mL, respectively. The most resistant species were *L. monocytogenes* and *S. enterica* with MIC and MBC values of 12.65 and 16.87 mg/mL, respectively, for both. These values varied from 32- to 63-fold higher than MIC and from 22- to 56-fold higher than MBC of controls.

**Table 2:** Minimum inhibitory (MIC) and minimum bactericidal (MBC) concentrations of *Baccharis dracunculifolia* aerial part essential oil and streptomycin and ampicillin controls.

Bacterium	Essential oil (mg/mL)		Streptomycin (mg/mL)		Ampicillin (mg/mL)	
	MIC	MBC	MIC	MBC	MIC	MBC
*Bacillus cereus*	1.1 ± 0.1^bB^	1.5 ± 0.02^aC^	0.10 ± 0.003^bA^	0.20 ± 0.06^bA^	0.25 ± 0.04^aC^	0.40 ± 0.03^aB^
*Enterobacter cloacae*	6.32 ± 0.6^dB^	8.43 ± 0.4^cB^	0.20 ± 0.02^cA^	0.30 ± 0.04^cA^	0.25 ± 0.03^aA^	0.50 ± 0.04^aC^
*Escherichia coli*	6.32 ± 0.9^dB^	8.43 ± 0.8^cB^	0.20 ± 002^cA^	0.30 ± 0.01^cA^	0.40 ± 0.02^bC^	0.50 ± 0.06^aC^
*Listeria monocytogenes*	12.65 ± 0.9^eB^	16.87 ± 1.0^dB^	0.20 ± 0.03^cA^	0.30 ± 0.00^cA^	0.40 ± 0.02^bC^	0.50 ± 0.02^aC^
*Micrococcus flavus*	3.15 ± 0.3^cB^	4.20 ± 0.6^bB^	0.20 ± 0.03^cA^	0.30 ± 0.01^cA^	0.25 ± 0.06^aA^	0.40 ± 0.09^aA^
*Pseudomonas aeruginosa*	1.05 ± 0.2^bC^	2.1 ± 0.6^aB^	0.20 ± 0.002^cA^	0.30 ± 0.00^cA^	0.75 ± 0.03^cB^	1.20 ± 0.20^cC^
*Salmonella enterica*	12.65 ± 0.6^eB^	16.87 ± 0.5^dB^	0.25 ± 0.02^cA^	0.50 ± 0.02^dA^	0.40 ± 0.02^bC^	0.75 ± 0.02^bC^
*Staphylococcus aureus*	0.5 ± 0.08^aC^	2.1 ± 0.5^aC^	0.04 ± 0.002^aA^	0.10 ± 0.002^aA^	0.25 ± 0.06^aB^	0.40 ± 0.01^aB^

*The averages followed by the same letters in the same columns (small letters) and in the same line (capital letters) for MIC or MBC do not differ by Tukey’s HSD test (p < 0.05).*

Minimum inhibitory concentration values of the essential oil against fungi ranged from 8.43 to 16.87 mg/mL, and the controls bifonazole from 0.10 to 0.20 mg/mL and ketoconazole from 0.20 to 2.5 mg/mL (Table 3). MFC values of the essential oil varied from 16.87 to 33.60 mg/mL and the controls bifonazole from 0.20 to 0.25 mg/mL and ketoconazole from 0.30 to 3.5 mg/mL (Table 3). MIC and MFC values of the essential oil against fungi were similar, from 8.43 to 16.87 mg/mL, respectively; only *A. niger* had MIC value of 16.87 mg/mL and MFC of 33.60 mg/mL, and *A. fumigatus* presented MIC of 12.65 mg/mL (Table 3). MIC and MFC values for the essential oil varied for MIC from 3- to 169- fold higher and MFC from 5- to 168-fold higher than the controls.

**Table 3:** Minimum inhibitory (MIC) and minimum fungicidal (MFC) concentrations of *Baccharis dracunculifolia* aerial part essential oil and bifonazole and ketoconazole controls.

Fungus	Essential oil (mg/mL)g		Bifonazole (mg/mL)		Ketoconazole (mg/mL)	
	MIC	MFC	MIC	MFC	MIC	MFC
*Aspergillus fumigatus*	12.65 ± 0.6^bC^	16.87 ± 0.8^aB^	0.15 ± 0.03^abA^	0.20 ± 0.03^aA^	0.20 ± 0.02^aA^	0.50 ± 0.3^aB^
*Aspergillus niger*	16.87 ± 1.0^cB^	33.60 ± 2.1^bC^	0.15 ± 0.04^abA^	0.20 ± 0.06^aA^	0.20 ± 0.02^aA^	0.50 ± 0.01^aB^
*Aspergillus versicolor*	8.43 ± 0.6^aC^	16.87 ± 0.5^aC^	0.10 ± 0.02^aA^	0.20 ± 0.03^aA^	0.20 ± 0.06^aB^	0.50 ± 0.02^aB^
*Aspergillus ochraceus*	8.43 ± 0.3^aC^	16.87 ± 0.9^aC^	0.15 ± 0.03^abB^	0.20 ± 0.02^aA^	1.50 ± 0.20^cA^	2.00 ± 0.30^bB^
*Penicillium funiculosum*	8.43 ± 0.6^aB^	16.87 ± 0.8^aC^	0.20 ± 0.01^bA^	0.25 ± 0.06^aA^	0.20 ± 0.02^aA^	0.50 ± 0.06^aB^
*Penicillium ochrochloron*	8.43 ± 0.6^aC^	16.87 ± 0.9^aC^	0.20 ± 0.01^bA^	0.25 ± 0.06^aA^	2.50 ± 0.30^dB^	3.50 ± 0.60^cB^
*Penicillium verrucosum*	8.43 ± 0.8^aB^	16.87 ± 1.1^aC^	0.10 ± 0.02^aA^	0.20 ± 0.03^aA^	0.20 ± 0.03^aA^	0.30 ± 0.02^aB^
*Trichoderma viride*	8.43 ± 0.9^aB^	16.87 ± 1.1^aC^	0.15 ± 0.03^abA^	0.20 ± 0.00^aA^	1.00 ± 0.10^bA^	1.00 ± 0.20^bB^

*The averages followed by the same letters in the same columns (small letters) and in the same line (capital letters) for MIC or MFC do not differ by Tukey’s HSD test (p < 0.05).*

## Discussion

The physical and chemical characteristics and the yield of *B. dracunculifolia* essential oil obtained in our study were different from the ones reported by Fabiane et al. (2008). For these authors, *B. dracunculifolia* leaf essential oil had yield of 1.5%, density of 91 g/mL, and refraction index of 1.4593. The differences may be related to the utilized plant part in the extraction of the essential oil. In our study, aerial parts were used and harvested during the flowering period when there is a natural increase in the production of the plant essential oil to attract pollinators. In the report by Fabiane et al. (2008) only leaves harvested during the vegetative phase of the plant were utilized. The plant phenophase is a determining factor in the yield, chemical composition, physical and chemical characteristics of the essential oil (de Sousa et al., 2009).

Different major compounds of *B. dracunculifolia* essential oil have been reported (Table 4). Frizzo et al. (2008) identified seventy components in *B. dracunculifolia* aerial part essential oil from Brazil, Uruguay and Bolivia. The identified components accounted for 91.4 to 97.2% of the total essential oil compositions. For these authors, *B. dracunculifolia* essential oils from different origins were quite different in quantitative and qualitative composition. The Brazilian essential oils were characterized by high contents of (*E*)-nerolidol, whereas the Uruguayan essential oils showed a predominance of viridiflorol with absence of (*E*)-nerolidol, and the Bolivian essential oils had higher contents of γ-cadinene, δ-cadinene, *t*-cadinol, and α-cadinol. Spathulenol was also identified in most *B. dracunculifolia* essential oils (Table 4).

**Table 4:** Major compounds of *B. dracunculifolia* essential oils.

Compound	Amount (%)	Source
Bicyclogermacrene	0.7–7.5	Frizzo et al., 2008
Caryophyllene	0.4–6.5	
Limonene	1.2–13.2	
Nerolidol	24.9	
Spathulenol	11.7	
β-Pinene	3.5–43.4	
Bicyclogermacrene	19.2	Massignani et al., 2009
Germacrene-D	21.5	
Nerolidol	23.6	
Spathulenol	6.0	
δ-Cadinene	3.6	
Spathulenol	9.5	Fabiane et al., 2008
Limonene	10.7	
Nerolidol	14.0	
β-Pinene	27.4	
β-Elemene	53.3	Lago et al., 2008
β-Pinene	19.6	Loayza et al., 1995
δ-Cadinene	15.9	
Nerolidol	23.6	Klopell et al., 2007

The differences found in *B. dracunculifolia* accesses make the importance of the essential oil chemical characterization in a bioassay evident. The chemical composition of the plant essential oil depends on the species, climate conditions, soil type, harvesting seasons, age of leaves, geographic region, and utilized extraction process. Seasonal variation is one of the main factors that affect the composition of essential oils (Chan et al., 2016). A study conducted by de Sousa et al. (2009) showed that the concentration of *trans*-nerolidol in *B. dracunculifolia* leaves was fivefold higher in March, 2005 (136.53 mg/100 g of plant) than that in July, 2004 (25.03 mg/100 g of plant).

Spathulenol (21,36%), the major compound of *Eugenia calycina* leaf essential oil showed antimicrobial activity against anaerobic bacteria *Prevotella nigrescens* and *Porphyromonas gingivalis* with MIC of 100 μg/mL (Sousa et al., 2015). Spathulenol (23.8%) and caryophyllene (14.9%), the major compounds of essential oil of *Salvia cilicica*, showed antimicrobial activity against *Mycobacterium tuberculosis*, *Microsporum gypseum*, *Trichophyton mentagrophytes*, and *Candida* spp. (Tan et al., 2016). Nerolidol, an isolated compound from Japanese cypress (*Chamaecyparis obtusa*), showed antifungal activity (MIC of 20 mg/mL) against *Microsporum gypseum in vitro* and *in vivo* with clinical remission of dermatomycoses (Lee et al., 2007). Nerolidol is the main component of many plants with antimicrobial activity (Chan et al., 2016). Thus, *B. dracunculifolia* essential oil from our study, whose major compounds were spathulenol (27.43%) and nerolidol (23.06%), is a promising alternative to control several microorganisms.

Our studies showed that *B. dracunculifolia* essential oil presents bactericidal activity, mainly against *S. aureus*, *B. cereus* and *P. aeruginosa*, important pathogenic agents. *S. aureus* is a pathogenic gram-positive bacterium with high infection and mortality rates (Kong et al., 2016). *P. aeruginosa* is a pathogenic Gram-negative agent that is resistant to several medications and cause infections in the bloodstream (Piao and Yuan, 2017). *B. cereus* is a Gram-positive bacterium that causes gastrointestinal infections and that may result in osteomyelitis, meningitis, and pneumonia (Malik-Tabassum et al., 2017), inflammation and eyesight loss (Callegan et al., 2017).

Minimum inhibitory concentration and MBC values found in our study were higher for the essential oils than for the controls. However, Van Vuuren (2008), in a review of over 500 publications detailing the antimicrobial activity of plants, proposed that essential oils with MIC of 2 mg/mL or lower or natural products with MIC values below 1 mg/mL could be considered noteworthy; however, in extracts, they must have MIC values below 8 mg/mL to be considered. Therefore, *B. dracunculifolia* essential oil of our study with MIC lower than 2 mg/mL (*S. aureus*, *B. cereus*, and *P. aeruginosa*) is considered noteworthy to control microorganisms.

The main compounds of *B. dracunculifolia* essential oil found in our study were oxygenated sesquiterpenes such as spathulenol and nerolidol that have high hydrophobicity, thereby allowing easier penetration across the plasma membrane and interaction with intracellular proteins and/or intra-organelle sites (Chan et al., 2016). The surface polarity of spathulenol and nerolidol is 20.2 Å^2^ and these compounds have one hydrogen bond donor and one acceptor (National Center for Biotechnology Information [NCBI]. PubChem Compound Database, 2018). Pajouhesh and Lenz (2005) reported that drugs with a PSA of 60 Å^2^ or less are completely absorbed by the cell, whereas those with at least 140 Å^2^ are not. Therefore, spathulenol as well as nerolidol are able to interact and pass through the cellular membrane.

For sesquiterpenes such as spathulenol and nerolidol, the antimicrobial action via cell membrane-disrupting mechanism is described and, hence, results in the leakage of K^+^ ions from bacterial cells (Inoue et al., 2004). According to Togashi et al. (2007) terpene alcohols with carbon chains of C10 to C12, as nerolidol, exhibit a strong antibacterial activity against *S. aureus*. As reported by Chan et al. (2016) besides causing membrane disruption, nerolidol can be responsible for the down-regulation of α-hemolysin gene *hla* expression in *S. aureus* determined via quantitative real-time PCR analyses (Lee et al., 2014). Chan et al. (2016) suggested that nerolidol is a therapeutic option to the development of drug combinations for antibacterial treatment, particularly against *S. aureus* and for multi-drug resistant bacteria.

The essential oil presented fungistatic and fungicidal action against *Aspergillus*, *Penicillium*, and *Trichoderma* genera. *Aspergillus* spp. are related to allergic reactions, respiratory problems and pulmonary infections (Yu et al., 2016). *A. ochraceus* is related to ochratoxin A production with pathogenic effects in animals and possible human carcinogen (Malir et al., 2016). *T. viride* causes adverse effects to health including respiratory problems (Larsen et al., 1996). *P. verrucosum* commonly grows on stored cereals (Bui-Klimke and Wu, 2015) and may produce ochratoxin A (Kuiper-Goodman and Scott, 1989) and citrinin (nephrotoxic) (Larsen et al., 2001). Thus, the identification of alternative compounds to control this fungus is relevant to manage damages caused by these fungi for food preservation, mainly with the increase in resistance to conventional chemical products.

Koul et al. (2008) warned that the uses of synthetic fungicides – in the post-harvest treatments of vegetables – could develop resistant fungal strains and indicated bio-based essential oils as fungicides. Sivakumar and Bautista-Baños (2014) recommend the use of essential oils to preserve food and Burt (2004) recommend concentrations from 0.1 to 6%. The fungicidal concentrations of *B. dracunculifolia* essential oil reported in our study range from 16.87 to 33.60 mg/mL (equivalent to 1.6 to 3.3%, m/v, respectively). Thus, the values found in our study are within the concentration of essential oils used for food preservation (Sivakumar and Bautista-Baños, 2014), which makes the essential oil from our study an alternative to develop applications in food preservation.

The utilization of essential oils to preserve food and as flavoring agents is considered to be Generally Recognized as Safe (GRAS) (Burt, 2004; Sivakumar and Bautista-Baños, 2014). In addition, the essential oil compounds such as carvacrol, carvone, cinnamaldehyde, citral, *p*-cymene, eugenol, limonene, menthol, and thymol are indicated with no risk to the consumer’s health. Also, there is no restriction for spathulenol and nerolidol, the major compounds of *B. dracunculifolia* essential oil described in our study.

One of the challenges to use essential oils to preserve food is the high cost that can be up to six times higher than chemical fungicides (Kouassi et al., 2012). However, Burt (2004) reported that the increase in the demand of essential oils can result in bioengineering of their synthesis in plants. Also, large scale production could reduce production cost. According to the European Pharmacopoeia, to develop the applications of essential oils, a minimum of 2 mL/kg of plants at flowering shoots is required (Nemeth and Bernath, 2008). Our study presents high yield (17 mL/kg dry plant) of *B. dracunculifolia* essential oil, increasing its potential utilization to control microorganisms.

## Conclusion

The aerial parts of *B. dracunculifolia* present high essential oil yield (17 mL/kg dry plant) with 30 components, and the major ones are spathulenol and nerolidol, followed by heptacosane, β-pinene, bicyclogermacrene, (*E*)-caryophyllene and germacrene D. The essential oil presents greater action against bacteria than against fungi, mainly against *S. aureus*, *B. cereus*, and *P. aeruginosa*. Essential oil of *B. dracunculifolia* may be a potential alternative to food applications in order to reduce synthetic chemicals in a more sustainable food industry.

## Author Contributions

GL, NC, and ZG conceived of the presented study. GL and ZG developed the theory and verified the analytical methods. LC obtained the essential oil and produced the fractions. JGo determined the chemical composition of the oil and fraction. JGl and MS investigated antimicrobial activities and with NC interpreted the results in the discussion section about antimicrobial activity. JGl, MS, and JGo contributed to the interpretation of the results. LC wrote the manuscript with support of GL, NC, and ZG. The final version of the manuscript was discussed, rewritten, and approved by all authors before submitting to the Journal.

## Conflict of Interest Statement

The authors declare that the research was conducted in the absence of any commercial or financial relationships that could be construed as a potential conflict of interest.
